# cDNA Isolation and Functional Characterization of UDP-d-glucuronic Acid 4-Epimerase Family from *Ornithogalum caudatum*

**DOI:** 10.3390/molecules21111505

**Published:** 2016-11-09

**Authors:** Sen Yin, Yu-Jia Sun, Ming Liu, Li-Na Li, Jian-Qiang Kong

**Affiliations:** State Key Laboratory of Bioactive Substance and Function of Natural Medicines & Ministry of Health Key Laboratory of Biosynthesis of Natural Products, Institute of Materia Medica, Chinese Academy of Medical Sciences & Peking Union Medical College, Beijing 100050, China; senyin@126.com (S.Y.); sunnyyujia@imm.ac.cn (Y.-J.S.); mingliu@imm.ac.cn (M.L.); lena699@163.com (L.-N.L.)

**Keywords:** UDP-d-glucuronic acid 4-epimerase, UDP-d-galacturonic acid, UDP-d-glucuronic acid, polysaccharides, *Ornithogalum caudatum*

## Abstract

d-Galacturonic acid (GalA) is an important component of GalA-containing polysaccharides in *Ornithogalum caudatum*. The incorporation of GalA into these polysaccharides from UDP-d-galacturonic acid (UDP-GalA) was reasonably known. However, the cDNAs involved in the biosynthesis of UDP-GalA were still unknown. In the present investigation, one candidate UDP-d-glucuronic acid 4-epimerase (UGlcAE) family with three members was isolated from *O. caudatum* based on RNA-Seq data. Bioinformatics analyses indicated all of the three isoforms, designated as OcUGlcAE1~3, were members of short-chain dehydrogenases/reductases (SDRs) and shared two conserved motifs. The three full-length cDNAs were then transformed to *Pichia pastoris* GS115 for heterologous expression. Data revealed both the supernatant and microsomal fractions from the recombinant *P. pastoris* expressing *OcUGlcAE3* can interconvert UDP-GalA and UDP-d-glucuronic acid (UDP-GlcA), while the other two OcUGlcAEs had no activity on UDP-GlcA and UDP-GalA. Furthermore, expression analyses of the three epimerases in varied tissues of *O. caudatum* were performed by real-time quantitative PCR (RT-qPCR). Results indicated *OcUGlcAE3*, together with the other two *OcUGlcAE*-like genes, was root-specific, displaying highest expression in roots. OcUGlcAE3 was UDP-d-glucuronic acid 4-epimerase and thus deemed to be involved in the biosynthesis of root polysaccharides. Moreover, *OcUGlcAE3* was proposed to be environmentally induced.

## 1. Introduction

**Scheme 1:**
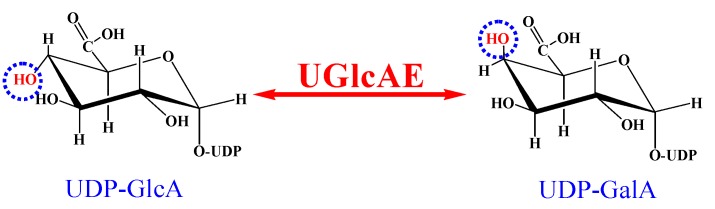
The interconversion of UDP-GlcA and UDP-GalA catalyzed by UglcAEs.

UDP-d-glucuronic acid 4-epimerase, a very specific membrane-bound 4-epimerase, is able to catalyze the interconversion of UDP-GlcA and UDP-GalA (Scheme 1) [1,2,3]. The resultant UDP-GalA, the activated nucleotide sugar form of GalA, can serve as the precursor of various bacterial polysaccharides and plant pectins [3,4,5]. Also, UDP-GalA is the precursor of health-promoting galacturonides, providing GalA component for the synthesis of galacturonides [6,7,8,9]. By this token, UGlcAE played crucial role in the synthesis of GalA-containing molecules with potent activities. Therefore, many attentions had been directed to the study of UGlcAE. Thus far, UGlcAE activities had been detected in diverse prokaryotes including *Bacillus cereus* [4], *Klebsiella pneumonia* [10], *Sinorhizobium meliloti* [11], *Escherichia coli* [12] and *Streptococcus pneumonia* [13]. However, relatively less information was available on cDNA isolation and functional characterization of genes encoding UGlcAE from plants, especially in monocots. Actually, the biochemical properties of UGlcAE from different organisms varied differentially. For example, some UGlcAE displayed substrate promiscuity [10], while the others were substrate specific [3,4,5,14,15]. Even in plant species, UGlcAEs in Arabidopsis differ from counterparts in *Poaceae species* [3,5,14,15]. These results together indicated our understanding toward UGlcAE was far from enough. It is therefore necessary to strengthen the study of UGlcAE. Moreover, characterization of the gene encoding UGlcAE is also a prerequisite to microbial UDP-GlcA production by synthetic biology and genetic engineering. Hence, an UGlcAE family containing three members was isolated from *Ornithogalum caudatum* in the present investigation.

*O. caudatum* is a monocot containing four anti-cancer polysaccharides, in which d-GalA is one of the main monosaccharides [16]. It was well known that the incorporation of d-GalA into these polysaccharides required the addition from its activated form UDP-GalA, which was synthesized from UDP-GlcA by UGlcAE. Accordingly, *UGlcAE* was deemed to be highly expressed in *O. caudatum*, which was in turn chosen as the start material for cDNA isolation of *UGlcAE*.

Here, three members of UGlcAE family, namely *OcUGlcAE1*, *OcUGlcAE2* and *OcUGlcAE3*, were isolated from *O. caudatum* based on the RNA-Seq data. In vitro enzymatic reaction verified the interconversion function between UDP-GlcA and UDP-GalA of OcUGlcAE3. Importantly, their roles in GalA-containing polysaccharides biosynthesis were also discussed based on real-time quantitative PCR (RT-qPCR).

## 2. Results and Discussion

### 2.1. Unigenes Retrieval from O. caudatum RNA-Seq Data

Transcriptome sequencing of *O. caudatum* was performed previously for the purpose of genes isolation [17,18,19]. The resultant RNA-Seq data was thus aligned for functional annotation by Blast X against public protein database deposited in GenBank library in the present investigation. Six unigenes showing high similarity with UGlcAE, namely unigenes 26961, 32208, 57762, 34210, 61748 and 61778, were retrieved (Table 1). Further ORF (open reading frame) Finder analyses toward these unigenes showed unigene 57762 contained a complete ORF encoding UGlcAE, while the other five unigenes harbored partial UGlcAE–encoding sequence. Specifically, unigene 57762 was 2247 bp long with an ORF of 1398 bp encoding 465 aa and consisted of a partial 5’-untranslated region (UTR) of 270 bp and 3’-UTR of 579 bp. Unigenes 26961 and 61778 were 1011 and 478 bp long, respectively. Both of them displayed similarity to 5’-end of an *UGlcAE* gene. Unigenes 32208 and 61748 were 634 and 664 bp in length, both exhibiting sequence identity to 3’-end of an *UGlcAE* gene. Unigene 34210 was 383 bp long and showed sequence similarity to a middle region of an *UGlcAE* gene. These data together revealed that there were at least three members in *O. caudatum* UGlcAE family. The detailed sequence information of the six unigenes was summarized in Table 1.

### 2.2. cDNA Isolation of OcUGlcAE Family

**Figure 1:**
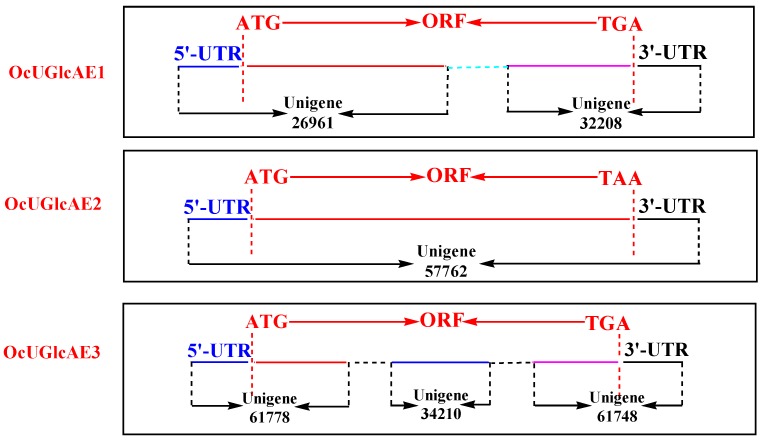
Schematic diagram of OcUGlcAE family in *O. caudatum*.

To verify the authenticity of these unigenes, PCR amplification of these sequences from *O. caudatum* by a nested PCR assay using gene-specific primers was performed. Unigenes 26961 and 32208 displayed high similarity with 5’-end and 3’-end of an *UGlcAE* gene respectively, suggesting the two unigenes were likely to locate on the same *UglcAE* gene. To test this notion, the 5’-end primers of unigene 26961 were paired with the 3’-end primers of unigene 32208 to amplify an *UglcAE* gene using *O. caudatum* cDNA as the template. After two rounds PCR, a specific PCR fragment, designated as OcUGlcAE1, was thus generated and ligated into *pEASY*^TM^-Blunt to yield the recombinant plasmid pEASY-OcUGlcAE1 for sequencing. Results showed that the amplified 1320 bp fragment showed 100% sequence identity with unigenes 26961 and 32208, suggesting the cDNA was a *bona fide UGlcAE* gene in *O. caudatum* genome (Figure 1).

The full-length ORF corresponding to unigene 57762 was directly isolated from *O. caudatum*. The resultant cDNA was 1398 bp in length and was designated to *OcUGlcAE2*, which were then inserted into *pEASY*^TM^-Blunt to generate a recombinant plasmid pEASY-OcUGlcAE2 for sequencing. Sequencing results indicated that the cDNA was identical to the unigene 577629 (Figure 1).

Unigenes 61778 and 61748 exhibited high similarity to the 5’- and 3’-end of an *UGlcAE* gene, which provided a clue that the two unigenes might be located in the same gene. Thus, the pair-wise primers corresponding to unigenes 61778 and 61748 were used to amplify the third *OcUGlcAE* gene from *O. caudatum*. The nested PCR led to the occurrence of a specific fragment of 1389 bp encoding 462 aa. This full-length ORF was then sequenced with the form of pEASY-OcUGlcAE3, a *pEASY*^TM^-Blunt derived plasmid. Result verified the 100% identity of *OcUGlcAE3* to unigenes 61778 and 61748. Interestingly, *OcUGlcAE3* was also 100% identical to unigene 34210. These data together revealed that unigenes 61778, 61748 and 34210 located on the same *OcUGlcAE3* gene. Therefore, there were at least three members in *O. caudatum* UGlcAE family (Figure 1). The three full-length *OcUGlcAE* genes were hence deposited in GenBank library with accession numbers of KX429689, KX429690 and KX429691 (Table 2).

### 2.3. Bioinformatics Characterization of OcUGlcAE Family

**Figure 2:**
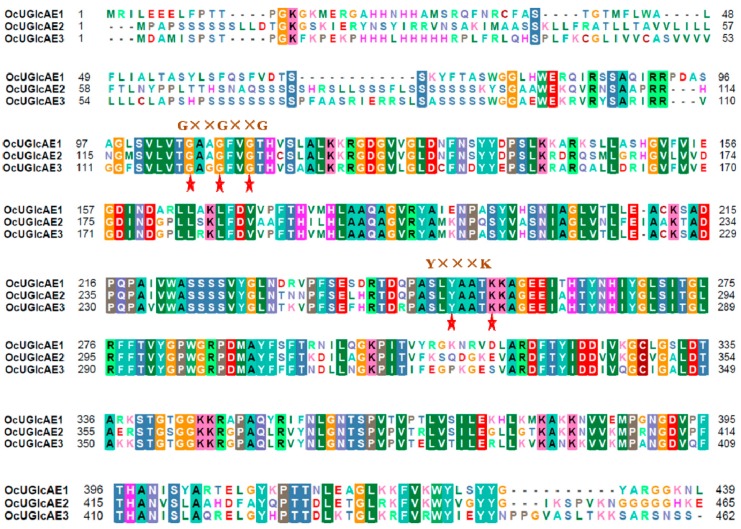
Sequence alignments of OcUGlcAE family. The red pentagram indicated the conserved motifs in OcUGlcAE family.

UGlcAE belongs to one member of the SDR enzyme families. Hence, the amino acid sequences of OcUGlcAE proteins contained two conserved motifs widely existed in SDR protein families. The first one was an N-terminal GxxGxxG motif (x = any amino acid) for the binding to NAD (P)^+^ (Figure 2). The second conserved motif referred to the catalytic YxxxK motif (where x represents any amino acid) (Figure 2).

Next, the most probable locations of trans-membrane helices in the three sequences were predicted by TMHMM program (http://www.cbs.dtu.dk/services/TMHMM/) [20]. As shown in the supporting material Figure S1, all the three proteins were trans-membrane proteins, which was consistent with the previous publications [3,14,15]. Among the three proteins, OcUGlcAE1 and OcUGlcAE3 both had two trans-membrane helices with more than 80% probability, while OcUGlcAE2 contained only one trans-membrane helice with more than 80% probability. Moreover, the region between 110 and 150 aa had about 35% probability to form the second trans-membrane helice in OcUGlcAE2 (Figure S1).

**Figure 3:**
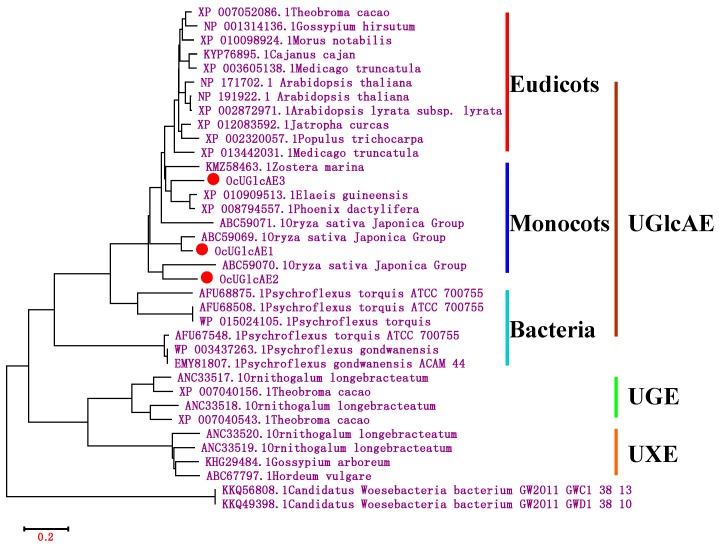
Phylogenetic tree of *O. caudatum* OcUGlcAE family with other UGlcAEs, UGEs and UXEs from varied organisms. The tree was constructed by MEGA 7.0 using Neighbor-joining method. The reliability of the tree was measured by bootstrap analysis with 1000 replicates. Red circles represent the member of OcUGlcAE family. Branch lengths are shown to scale.

**Figure 4:**
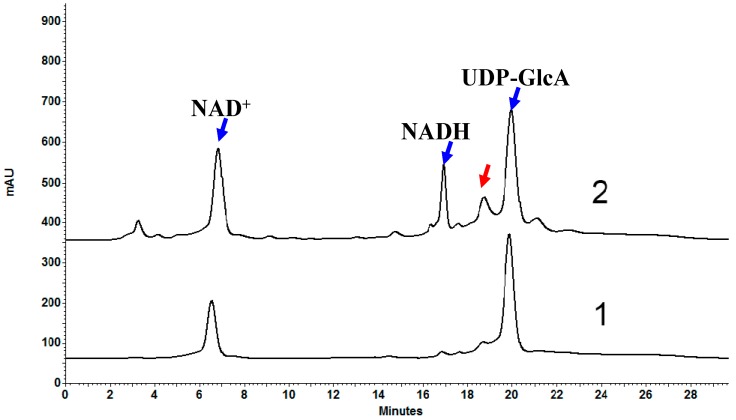
HPLC analyses of the products formed by the supernatant from *P. pastoris* expressing the empty vector alone (**1**) or pPIC3.5kOcUGlcAE3 (**2**) in the presence of UDP-GlcA and NAD^+^. The newly formed UDP-GalA was marked by a red arrow.

Phylogenetic relationships of OcUGlcAE with other UGlcAE, UGE (UDP-d-glucose 4-epimerase) and UXE (UDP-d-xylose 4-epimerase) enzymes were analyzed using the neighbor-joining method (Figure 3). As indicated in Figure 4, UGlcAE, UGE and UXE belonged to three distinct clades, suggesting the divergent functions among the tree enzymes were present through evolution. The UGlcAE family could be divided into three groups, which included diverse UGlcAEs from eudicots, monocots and bacteria, respectively. Although OcUGlcAE1~3 were most similar to monocots UGlcAE, the three UGlcAE enzymes belonged to different subgroups (Figure 3). The evolutionary differences among the three OcUGlcAE enzymes may provide a clue that the three enzymes functioned differentially.

### 2.4. Bacterial Expression of OcUGlcAEs

The three OcUGlcAE enzymes were predicted to be membrane-bound proteins by TMHMM algorithm (Figure S1). Recombinant membrane-associated proteins were shown to be difficult to express and purify in *E. coli*, presumably due to membrane-spanning domains [17,21]. The truncated OcUGlcAEs, lacking the putative trans-membrane motifs, were thus generated for soluble expressions in *E. coli*. Specifically, the trans-membrane domains of the three proteins were deleted from the N-terminus to form three truncated versions, namely OcUGlcAE1 (Δ1–116), OcUGlcAE2 (Δ1–136) and OcUGlcAE3 (Δ1–128). Then, these N-terminal truncated *OcUGlcAE* genes were inserted into *Eco*RI/*Hin*d III linearized pET-28a (+) to yield three recombinant plasmids, pET28aOcUGlcAE1 (Δ1–116), pET28aOcUGlcAE2 (Δ1–136) and pET28aOcUGlcAE3 (Δ1–128) (Table S1). Truncated OcUGlcAE proteins (trun-OcUGlcAEs) were then induced to be heterologously expressed in *Trans*etta (DE3) with the form of pET28aOcUGlcAE1 (Δ1–116), pET28aOcUGlcAE2 (Δ1–136) and pET28aOcUGlcAE3 (Δ1–128), respectively. SDS-PAGE analyses showed these truncated proteins were mainly appeared as insoluble inclusion bodies, suggesting the improper folding of these trun-OcUGlAE proteins occurred. It was well known that molecular chaperones were able to assist the refolding of inclusion body [22,23]. Hence, a chaperone plasmid pGro7 was co-expressed with each of the three plasmids containing truncated *OcUGlcAE* genes in *E. coli*. The chaperone plasmid pGro7 contained two genes, *groES* and *groEL*, encoding for chaperone proteins. GroES and GroEL were well known to work in cooperation in the folding process [22,23]. With the help the chaperone proteins GroES and GroEL, the recovery of the soluble trun-OcUGlcAE proteins from inclusion bodies increased, as shown in the supporting material Figure S2.

Next, these *E. coli* crude extract expressing trun-OcUGlcAE proteins were applied to examine their activities in a total reaction volume of 100 μL. After incubation at 30 °C for 30 min, twenty microlitres supernatant was directly analyzed by HPLC. As illustrated in Figure S3, there are no specific peaks corresponding reaction products in the three reaction mixtures, suggesting no activities toward UDP-GlcA occurred in these OcUGlcAE proteins (Figure S3). The unavailability of active recombinant enzymes was present when trun-OcUGlcAEs were expressed in *E. coli*, presumably due to the N-terminal truncation that resulted in improper folding and decreased stability of these recombinant proteins. Three full-length OcUGlcAE proteins were therefore heterologously expressed in *P. pastoris* for active recombinant enzymes.

### 2.5. Heterologous Expression of OcUGlcAEs in P. pastoris

**Figure 5:**
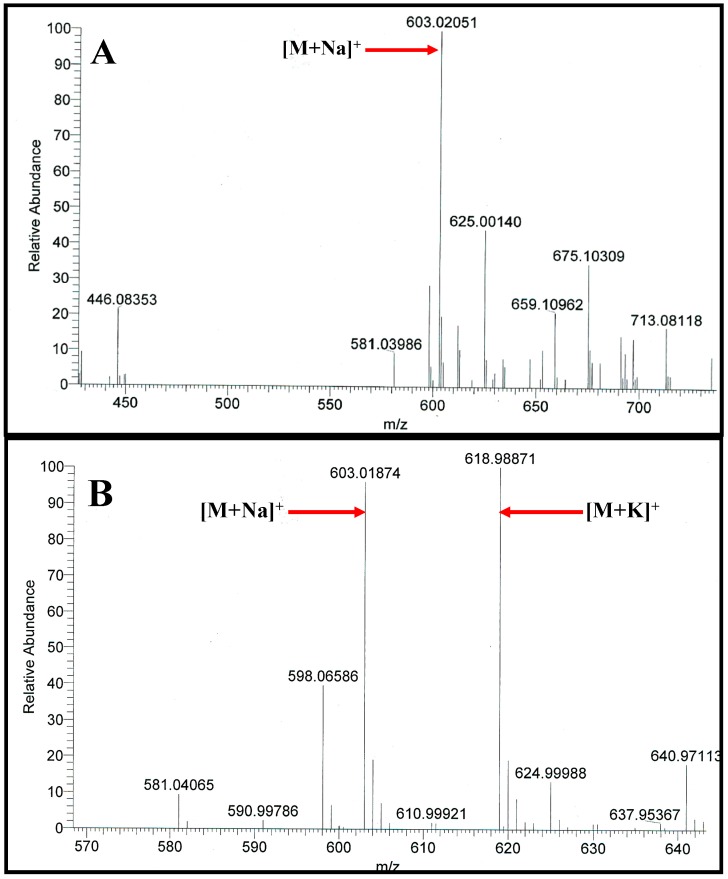
Electrospray positive ion mass spectra for UDP-GalA (**A**) and UDP-GlcA (**B**).

For analysis of enzymatic activity of OcUGlcAEs, the supernatant of three recombinant *P. pastoris* strains containing pPIC3.5kOcUGlcAEs was used directly as the biocatalyst for the interconversion of UDP-GalA and UDP-GlcA, respectively. The newly formed products were monitored by a HPLC-based assay. When UDP-GlcA and NAD^+^ were incubated with the supernatant harboring a recombinant OcUGlcAE3 protein, a new peak, having the same retention time and UV spectra as the authentic UDP-GalA, was detected in the HPLC profile (Figure 4). The supernatant of *P. pastoris* expressing the empty control vector did not yield a new peak, indicating the observed new peak was exclusively produced by the recombinant OcUGlcAE3. To further verify that the newly formed reaction product representing exactly UDP-GalA, the UDP-GalA standard was co-injected with the reaction mixture under the HPLC-based assay. Only a UDP-GalA peak with no shouldering was found by HPLC analysis, suggesting UDP-GalA was produced from 4-epimerization of UDP-GlcA (Figure S4). Finally, to determine the identity of the newly formed product, the enzymatic product eluting from the HPLC column was collected and confirmed by mass spectrometry. The mass spectrometer gave the most abundant ion at *m*/*z* 603.01874, corresponding to an adduct of the detected molecule with a sodium atom [M + Na]^+^. The molecular mass of the epimerized derivative of UDP-GalA catalyzed by OcUGlcA3 was thus inferred to be 580, which was identical to that of UDP-GalA standard (Figure 5A). Taken together, these data unambiguously identified that the newly formed product catalyzed by recombinant OcUGlcA3 proteins was UDP-GalA.

**Figure 6:**
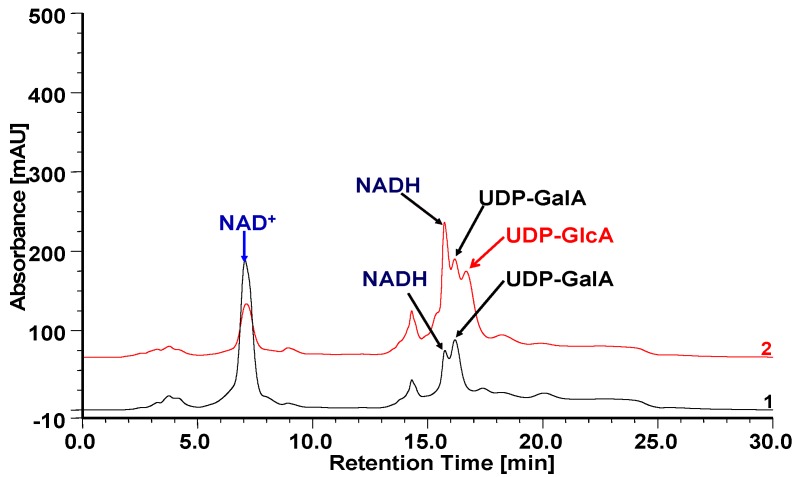
HPLC analyses of reaction mixture containing the supernatant from GS115 (**1**) or *P. pastoris* expressing OcUGlcAE3 (**2**) using UDP-GalA as the substrate.

Next, the ability of the recombinant OcUGlcAE3 to convert UDP-GalA to UDP-GlcA was also confirmed. As shown in Figure 6, incubation of the supernatant from *P. pastoris* expressing OcUGlcAE3 with UDP-GalA resulted in the generation of UDP-GlcA, which was determined by co-injection analysis (Figure S5) and mass spectrometry (Figure 5B) as mentioned above.

**Figure 7:**
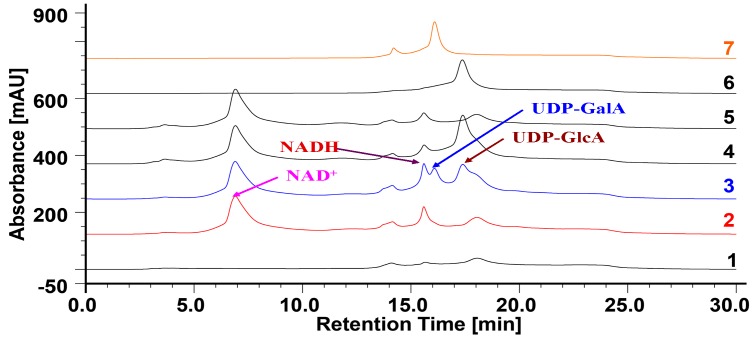
HPLC profile of reaction mixture catalyzed by microsome fractions from *P. pastoris*. **1**, HPLC profile of microsomes from *P. pastoris* expressing OcUGlcAE3. **2**, HPLC analysis of microsomes from *P. pastoris* expressing OcUGlcAE3 in the presence of NAD^+^. **3**, HPLC chromatograph of reaction mixture containing microsomes from *P. pastoris* expressing OcUGlcAE3 in the presence of UDP-GlcA and NAD^+^. **4**, HPLC profile of reaction mixture containing microsomes from *P. pastoris* expressing empty vector alone in the presence of UDP-GlcA and NAD^+^. **5**, HPLC analysis of microsomes from GS115 in the presence of NAD^+^. **6** and **7** refer to HPLC separation of the standard UDP-GlcA (**6**) and UDP-GalA (**7**), respectively.

Besides the supernatant, the microsomal fraction from *P. pastoris* expressing OcUGlcAE3 was observed to have 4-epimerase activity capable of interconverting UDP-GalA and UDP-GlcA (Figure 7). These data indicated that the recombinant OcUGlcAE3 can form microsomal protein by the N-terminal membrane anchor sequence, or can be secrete into the extracellular space through the guidance of the signal peptide. Moreover, OcUGlcAE3 did not require exogenous NAD^+^, suggesting the recombinant OcUGlcAE3 contained tightly bound NAD^+^, which was consistent with previous reports [3,5,15].

On the contrary, the 4-epimerase activity was not detected either in the supernatant or microsomal fraction from *P. pastoris* expressing OcUGlcAE1 or OcUGlcAE2.

### 2.6. Biochemical Properties of OcUGlcAE3

**Figure 8:**
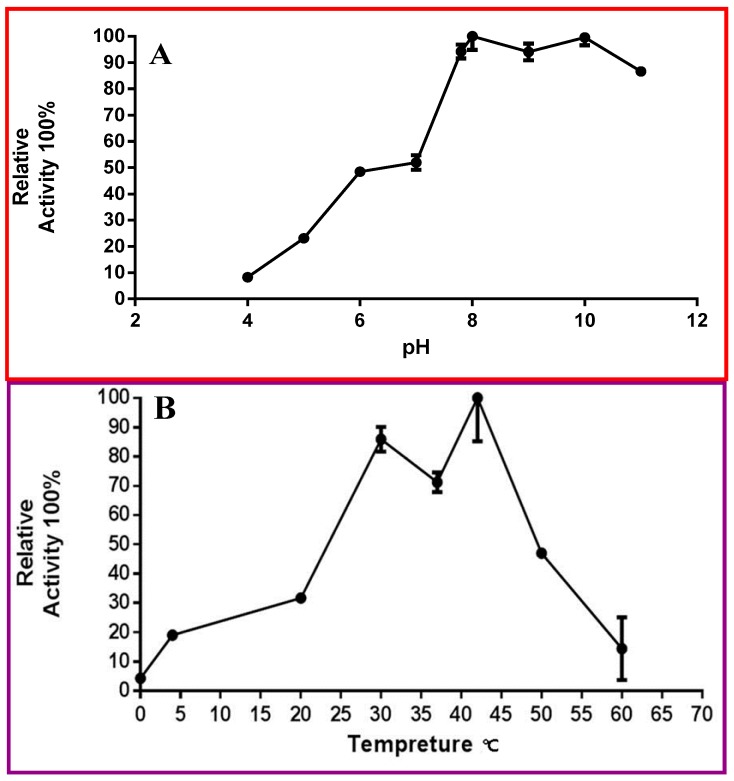
The effect of pH (**A**) or temperature (**B**) on the OcUGlcAE3 activity.

OcUGlcAE3 was active over a relatively broad pH range between 4 and 11 (Figure 8A). The pH value curve of OcUGlcAE3 was shown as an “M” type (Figure 8A). OcUGlcAE3 retained no more than 10% activity when the pH value was 4.0. The enzymatic activity increased gradually with the increase of pH. When pH reached the range between 8 and 8.5, the enzyme displayed the highest activity. With the further increase of pH to about 9, the activity of the enzyme decreased slightly. And then, as the pH increased from 9 to 10, the activity of OcUGlcAE3 increased gradually, and reached the highest activity again at pH value of 10. Next, with the further increase of pH, the activity of OcUGlcAE3 began to decrease. When pH reached 12, OcUGlcAE3 kept only 80% of its activity. The pH profile of OcUGlcAE3 showed an “M” type, which was inconsistent with that of other UGlcAEs [14,15]. Although the exact reason of this discrepancy was not clear, the alkaline preference of OcUGlcAE3 was certain, which was also observed in other UGlcAEs, like Arabidopsis UGlcAEs [3,14,15] and Poaceae UGlcAEs [5].

**Figure 9:**
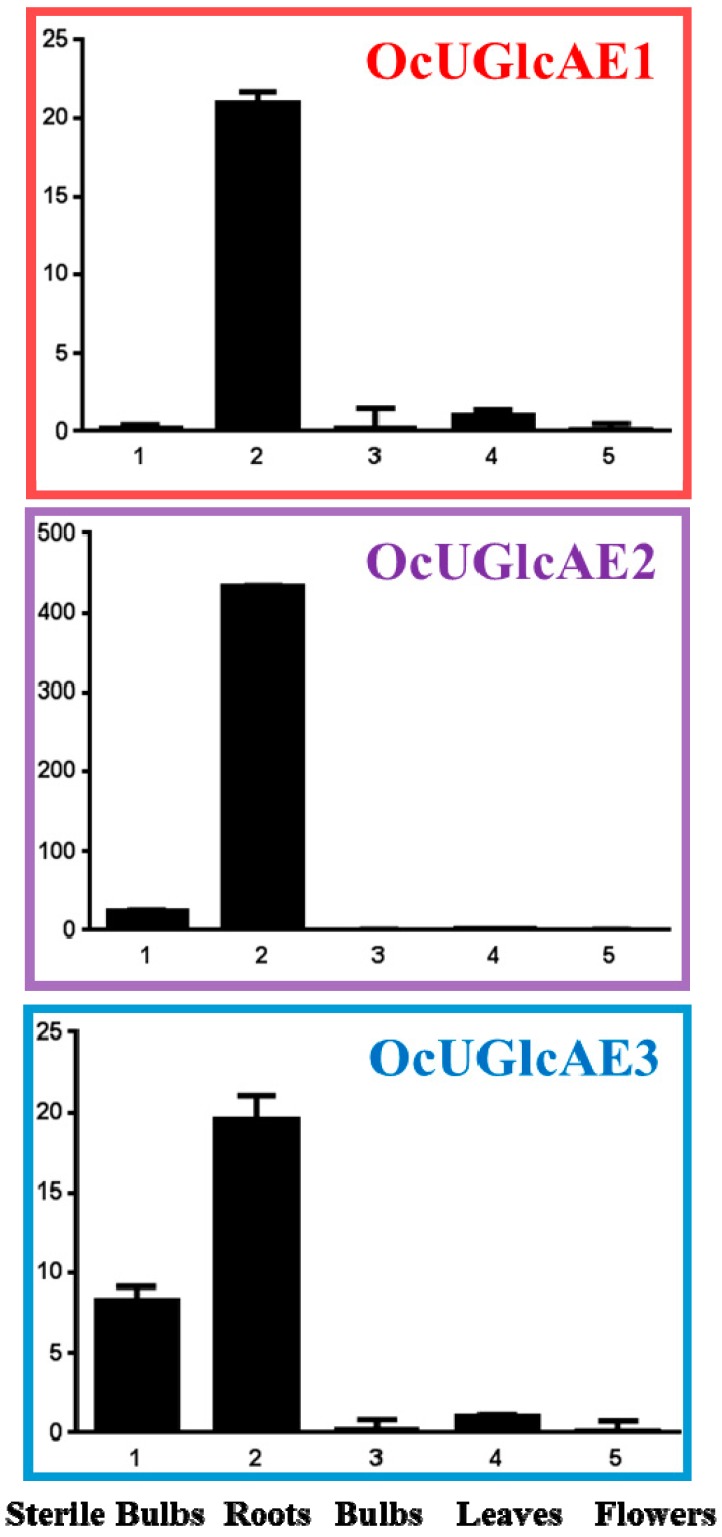
Expression profiles of the three *OcUGlcAE* genes in various *O. caudatum* tissues including sterile bulbs, roots, bulbs, leaves and flowers by RT-qPCR. Relative transcript levels were standardised to the constitute *GAPDH* gene expression. Data shown represent the average ± SD (standard deviation) of three independent experiments.

OcUGlcAE3 displayed activity over a broad temperature range between 0 °C and 60 °C and had maximal activity at 42 °C (Figure 9B). The temperature profile of OcUGlcAE also showed “M” type (Figure 8B). The activity of OcUGlcAE3 gradually rose with the increase of temperature from 0 to 30 °C. OcUGlcAE3 activity reached the second high level when the temperature was 30 °C. Then, with the further increase of temperature, the activity of OcUGlcAE3 began to decrease. When the temperature reached 37 °C, OcUGlcAE3 was only 70% activity. With the further increase of temperature, OcUGlcAE3 activity began to increase and reached the highest level at 42 °C. Next, as the temperature increased from 42 °C to 60 °C, the activity of OcUGlcAE3 decreased significantly. When the temperature reached 65 °C, OcUGlcAE2 only kept less than 10% activity (Figure 8B). Further kinetic analyses indicated that OcUGlcAE3 had an apparent *K*_m_ of 0.17 ± 0.07 mM for UDP-d-Glc, which was comparable with that of Arabidopsis UGlcAEs [3].

### 2.7. Expression Analyses of OcUGlcAE in O. caudatum

OcUGlcAE regulated the biosynthesis of UDP-GalA, a monosaccharide donor for the synthesis of GalA-containing polysaccharides in *O. caudatum*. OcUGlcAE was thus proposed to be responsible for the biosynthesis these GalA-containing polysaccharides. To probe the relevance of OcUGlcAE to polysaccharides biosynthesis, expression profiles of the three genes in varied tissues of *O. caudatum* were hence performed based on RT-qPCR results (Figure 9). The transcript level of *OcUGlcAE3* was most abundant in roots, where *OcUGlcAE3* mRNA levels were almost 2-fold higher than that in sterile bulbs of *O. caudatum* (Figure 9). This root-specific expression pattern of *OcUGlcAE3* revealed its involvement in the biosynthesis of root polysaccharides containing uronic acids. The sterile bulbs, cultivated in sterile 6,7-V medium under a controlled condition, were the same organ as *O. caudatum* bulbs, which were grown in pots under a natural environmental condition. Theoretically, the expressions of *OcUGlcAE3* in sterile bulbs and *O. caudatum* bulbs should be consistent. Actually, the transcript level of *OcUGlcAE3* in sterile bulbs was significantly higher than that in *O. caudatum* bulbs, indicating environmental conditions had an effect on the expression of *OcUGlcAE3* (Figure 9). Also, *OcUGlcAE1* and *OcUGlcAE*2 were also mainly expressed in roots. The expression levels of *OcUGlcAE1* and *OcUGlcAE2* were barely detected in bulbs, leaves and flowers (Figure 9).

### 2.8. Discussion

There were at least four GalA-containing polysaccharides, which exhibited potent anticancer activity against Sarcoma 180 solid tumor, in *O. caudatum* [16]. UDP-GalA was reasonably inferred to be the sole donor for these polysaccharides biosynthesis. However, the genes responsible for UDP-GalA biosynthesis in *O. caudatum* had never been documented. In this research, a small UGlcAE family with three candidate members was isolated from *O. caudatum* for the first time. In vitro reaction verified the activity of OcUGlcAE3, interconverting UDP-GlcA and UDP-GalA. Previous experiments showed that these polysaccharides extracted from the whole plant [16], indicating the root of *O. caudatum* might also contain these anti-cancer polysaccharides. The high expression of OcUGlcAE3 in the root was thus indicative of its involvement in the biosynthesis of GalA-containing polysaccharides in roots. Of course, all of this need to be further verified through experiments.

Although there had been few reports of the application of UGlcAEs in microbial production of GalA-containing molecules at present, the successful characterization of OcUGlcAE3 would no doubt provide a more choice for gene parts used for GalA-containing molecules production in engineered cell factories in the future. So far, the plant *UGlcAE* genes were mainly isolated from *Arabidopsis thaliana* [3,5,14,15], *Zea mays* [5] and *Oryza sativa japonica* [5]. Compared to these isoforms, the *K*_m_ value for UDP-GlcA of OcUGlcAE3 was relatively low, indicating its higher affinity to the substrate UDP-GlcA. Moreover, unlike AtUGlcAE1 from Arabidopsis, OcUGlcAE3 had a wider temperature tolerance [3].

In vitro functional identification of OcUGlcAE3 was carried out on the premise that the soluble recombinant OcUGlcAE3 should be yielded. It was expected that the active recombinant protein could not been detected when the full-length *OcUGlcAE3* gene was expressed in *E. coli* due to the presence of the trans-membrane domains in its N-terminus. Truncated mutant of OcUGlcAE3 was thus generated by a deletion of a putative trans-membrane domain of 128 aa in the N-terminus. Unexpectedly, no soluble expression of OcUGlcAE3 was induced to express in *E. coli*. This result was inconsistent with previous reports, where the soluble truncated UGlcAE enzymes from other organisms were obtained [3,5]. The reason for this discrepancy may be the length of amino acids deleted from the N-terminus of UGlcAEs. As illustrated in the supporting Table S2, there were one or two trans-membrane domains in varied UGlcAEs. The first domain is generally located between 30–70 aa from the N-terminus, while the second motif lay in the domain between 85–140 aa (Table S2). A large majority of UGlcAE proteins have the first domain, while only a few UGlcAE isoforms have both domains (Table S2). In previous reports, the truncated UGlcAE enzymes were obtained by a deletion of the first trans-membrane domain [3,5]. The expression of these truncated *UGlcAE* genes generally leads to soluble UGlcAE proteins. On the contrary, when truncated OcUGlcAE3 with a deletion of two trans-membrane domains were introduced into *E. coli* for expression, no soluble recombinant OcUGlcAE protein was obtained. These data collectively revealed the length of amino acids removed from the N-terminal of UGlcAEs had effect on the soluble expression. Moreover, the length of amino acids removed from UGlcAE enzymes also affects the activities of UGlcAE proteins. The truncated UGlcAEs with a deletion of no more than 72 aa in previous reports exhibited 4-epimerase activity, catalyzing the interconversion of UDP-GlcA and UDP-GalA [3,5]. However, the truncated OcUGlcAE3 had no action on UDP-GlcA. The truncated OcUGlcAE3 removed a stretch of 128 aa from its N-terminus, where the GxxGxxG motif located (Figure S6). On the contrary, those UGlcAEs with a truncation of no more than 72 aa kept the presence of GxxGxxG motif, which was as illustrated in Figure S6. GxxGxxG motif is a conserved domain that performs the function of binding NAD^+^. NAD^+^ is a cofactor which is required for UGlcAEs activity. The deletion of the conversed GxxGxxG motif in turn resulted in the activity loss of OcUGlcAE3. Together, these data indicated the N-terminus of UGlcAEs played important roles in their soluble expression and 4-epimerase activity.

## 3. Materials and Methods

### 3.1. Plasmids, Strains and Compounds

Plasmids *pEASY*^®^-Blunt (TransGen Biotech Co. Ltd., Beijing, China) and pET-28a (+) (Novagen, Madison, WI, USA) were used for gene amplification and heterologous expression, respectively. Integration plasmid pPIC3.5K (Invitrogen, Carlsbad, CA, USA) was functional in *Pichia* strain GS115.

*E. coli* strains *Trans*1-T1 and *Trans*etta (DE3) were obtained from TransGen Co. Ltd. and were used as a bacterial host for recombinant plasmids amplification and enzymes expression, respectively. *Pichia pastoris* GS115 was used as a eukaryotic host for heterologous expression of OcGlcAE1~3. The detailed plasmids and strains used in this study are provided in Table S1.

UDP-d-GalA was from CarboSource Services (University of Georgia, Athens, GA, USA). UDP-d-GlcA, NAD^+^ and NADH were obtained from Sigma-Aldrich Co. LLC (St. Louis, MO, USA). All other chemicals used in this study were of analytical grade.

### 3.2. Bacterial and Yeast Culture Media

*E. coli* strain was grown in Luria-Bertani medium (10 g/L Bacto-Tryptone, 5 g/L Bacto-yeast extract, 10 g/L NaCl) supplemented with appropriate antibiotics when required for selection. Yeast extract–peptone–dextrose (YPD) medium (2% (*w*/*v*) peptone, 1% (*w*/*v*) yeast extract, and 2% (*w*/*v*) dextrose) was used for yeast culture. Minimal dextrose (MD) medium (1.34% YNB, 4 × 10^−5^% biotin, 2% dextrose, 1.5% agar) was applied in selection of yeast His^+^ transformants. The buffered minimal glycerol-complex (BMGY) medium (100 mM potassium phosphate buffer (pH 6.0), 2% peptone, 1% yeast extract, 1.34% yeast nitrogen base with ammonium sulphate but without amino acids, 1% glycerol, and 4 × 10^−5^% biotin) was prepared for yeast protein induction.

### 3.3. Plant Material

*O. caudatum* plants were maintained in our experimental pots or sterile conditions as previously described [17,18]. *O. caudatum* tissues were collected as described by Li et al. [18] and used as the starting materials for cDNA isolation and RT-qPCR analyses of *OcUGlcAE*s.

### 3.4. Unigenes Retrieval from O. caudatum RNA-Seq Data

Transcriptome sequencing of *O. caudatum* was carried out previously [19,24]. The resulting massive unigenes were functionally annotated by Blast X against protein database deposited in GenBank library. Unigenes sharing highly similarity to UGlcAE were hence retrieved from RNA-Seq data of *O. caudatum*.

### 3.5. cDNA Isolation and Bioinformatics Analyses of OcUGlcAEs

To verify the authenticity of the retrieved unigenes, cDNA amplification was performed using gene-specific primers by nested PCR. The resulting cDNA sequences were then sequenced for their identity and further analyzed by bioinformatics tools in details as introduced by Li et al. [18]. A phylogenetic tree was constructed using the Neighbor-joining method with the molecular evolutionary genetics analysis (MEGA) software, version 7.0 [25]. The reliability of the tree was measured by bootstrap analysis with 1000 replicates. The primers used in this study were listed in supporting material Table S3.

### 3.6. Bacterial Expression of OcUGlcAEs

Three truncated *OcUGlcAE* genes were inserted into *Hin*dIII/*Eco*RI linearized pET-28a (+) to yield three recombinant plasmids by In-fusion technology. The primers used for plasmids construction were listed in Table S3. These pET-28a (+) derived plasmids were then transformed into *Trans*etta (DE3) for heterologous expression. The resultant *E. coli* crude extract was applied to perform SDS-PAGE analyses as described previously [17,18,19]. The enzyme activities of these OcUGlcAE proteins were examined in a total volume of 100 μL containing 5 μL UDP-GlcA (10 mM), 5 μL NAD^+^ (10 mM) and 30 μL crude protein extract. The mixture was incubated at 30 °C for 30 min. The reaction was then stopped by adding 25 μL chloroform. After vortex and removal of proteins by 0.45 µm membrane, twenty microliters supernatant was directly analyzed by HPLC.

### 3.7. Yeast Expression and Functional Characterization of OcUGlcAE1

Three full-length *OcUGlcAE* genes were ligated with *Bam*H I and *Eco*R I digested pPIC3.5K to generate three expression plasmids, pPIC3.5KOcUGlcAE1, pPIC3.5KOcUGlcAE2 and pPIC3.5KOcUGlcAE3. The three plasmids were then digested by *Sac*I and the resultant *Sac*I-linearized recombinant plasmids were transformed into *P. pastoris* by electroporation as described by the manufacturer.

Cells were spread on MD medium and incubated at 30 °C for 48–72 h until colonies formed. Several colonies were picked and re-inoculated in YPD plates containing increasing geneticin at a final concentration of 0, 2 and 4 mg/mL. The YPD plates were incubated at 30 °C for 2 to 3 days. The colonies resistant to 4 mg/mL geneticin were picked for further PCR confirmation using 5’AOX1 and 3’AOX1 primers (Table S3).

The positive *P. pastoris* transformants were grown in 15-mL volumes (50 mL baffled shake flasks) of BMGY medium at 30 °C with constant shaking (220 rpm) until an OD_600_ of 5–6 was obtained. The induction of OcUGlcAEs was performed for 120 h by daily supplementation of filter-sterilized 100% methanol at a final concentration of 1%. The yeast cells were then harvested by low speed centrifugation (1000 g, 10 min, 4 °C) and washed twice with ddH_2_O. All further steps were at 4 °C or on ice. The resultant pellets were re-suspended in phosphate buffer (pH 7.0) and subjected to be disrupted by high pressure (1200 bar, 6–8 times).The obtained homogenate was centrifuged at 12,000× *g* for 30 min to remove the cell debris. Next, the resultant supernatant at this stage was ultra-centrifuged at 160,000× *g* for 1.5 h to sediment the microsomes. The supernatant and microsomal fractions were used as the biocatalyst for interconversion of UDP-GlcA and UDP-GalA, respectively. The assay was performed in a total volume of 100 μL containing 5 μL UDP-GlcA (10 mM), 5 μL NAD^+^ (10 mM) and 30 μL supernatant. The mixture was incubated at 30 °C for 30 min. The reaction was then stopped by adding 25 μL chloroform. After vortex and removal of proteins by 0.45 µm membrane, the samples were analyzed by HPLC (high performance liquid chromatography) and LC-MS.

HPLC was performed on a Hitachi Elite Lachrom HPLC system (Hitachi, Tokyo, Japan) equipped with a diode array detector and Dionex CarboPac^TM^ PA10 carbohydrate column (4 × 250 mm, Thermo, Waltham, MA, USA). For generation of an analytical scale, the mobile phase consisted of ddH_2_O (mobile phase A) and acetic acid-ammonium acetate buffer (700 mM, pH 5.2, mobile phase B). The concentration gradient of mobile phase B was programmed as follows: 0–20 min, 5%; 20–32 min, 45%; 32–33 min, 100%; 33–40 min, 5%. The flow rate of the mobile phase was 1 mL/min. Detector signal was monitored at a wavelength of 261 nm. The column temperature was kept ambient and injection volume was 20 μL. LC-MS was performed as described by Li et al. [18].

### 3.8. Enzymatic Characterization of OcUGlcAE3

To obtain a comprehensive knowledge of OcUGlcAE3, the optimum pH and temperature evaluation and kinetic analyses of OcUGlcAE3 were performed as described previously with some modifications [17,18,26]. To determine the effect of pH on OcUGlcAE3, the supernatant from *P. pastoris* expressing OcUGlcAE3 was incubated with UDP-GlcA in sodium acetate-acetic acid buffer (pH 4) and phosphate buffered saline (PBS, pH 5–11) at 37 °C for 1 h. For determination of optimal temperature, reactions were conducted for 1 h in 100 mM PBS buffer (pH 7.8) between 0 °C and 60 °C with a 5 °C interval. These assays were carried out using UDP-GlcA as the substrate. The formation of UDP-GalA was monitored by HPLC. Each assay was performed in triplicate.

Kinetic analysis of OcUGlcAE3 was conducted in 200 μL PBS buffer (100 mM, pH 7.8) containing 30 μL supernatant from *P. pastoris* expressing OcUGlcAE3 and varied concentrations of UDP-GlcA in the range of 0.156–10 mM. These assays were performed at 37 °C for 20 min. Each assay was performed in triplicate. The product formation was monitored by HPLC at 259 nm. The rates of product formation were thus determined as a function of substrate concentrations. The kinetic values were thus calculated by double reciprocal plot (Lineweaver-Burk plot), which was constructed by Graphpad Prism 5.01 software.

### 3.9. Expression Analyses of OcUGlcAEs in O. caudatum

Expression analyses of *OcUGlcAE*s in varied tissues including roots, bulbs, leaves, flowers and sterile bulbs were performed using RT-qPCR technology described in our recent publications [17,18]. GAPDH (glyceraldehyde-3-phosphate dehydrogenase) was used as an internal control to normalize the expression level of *OcUGlcAE* genes. The gene-specific primers used for RT-qPCR assay were designed according to Primer Express 3.0 software (Applied Biosystems, Waltham, MA, USA) and summarized in Table S3.

## 4. Conclusions

UDP-GalA is an important donor for GalA-containing polysaccharides in *O. caudatum*. However, the genes responsible for the UDP-GalA biosynthesis had not been documented. In this investigation, a small UGlcAE family with three members was isolated from *O. caudatum* based on RNA-Seq data for the first time. *OcUGlcAE3* was then identified to be a *bona fide* gene by in vitro enzymatic action. RT-qPCR was thus applied to analyze the expression profile of *OcUGlAE3*. Data showed that the expression of *OcUGlcAE3* was root-specific and OcUGlcAE3 was hence deemed to be involved in the biosynthesis of root-specific GalA-containing polysaccharides. Moreover, *OcUGlcAE3* was proposed to be environmentally induced.

## Figures and Tables

**Table 1:** Unigenes sharing high sequence similarity with UDP-d-glucuronic acid 4-epimerase.

Unigene ID	Length (bp)	Sequence Structure (bp)		
		5’-UTR	ORF	3’-UTR
26961	1011	206	805 *	N
32208	634	N	459 *	175
57762	2247	270	1398	579
34210	383	N	383 *	N
61748	664	N	354 *	310
61778	478	148	330 *	N

N, no sequences; *, partial ORF sequence.

**Table 2:** Comparison of the predicted OcUGlcAE proteins as deduced from their cDNA sequences.

Nucleotide					Protein				
Gene	Accession Number	Corresponding Unigene (s)	Length (bp)	ORF (bp)	a.a.	kDa	p*I*	Identity (%)	
								OcUGlcAE2	OcUGlcAE3
*OcUGlcAE1*	KX429689	Unigenes 26961 and 32208	1701	1320	439	48.462	9.66	74	76
*OcUGlcAE2*	KX429690	Unigene 57762	2247	1398	465	50.057	9.76	100	77
*OcUGlcAE3*	KX429691	Unigenes 61778, 61748 and 34210	1847	1389	463	50.579	9.77	77	100

## Supplementary Materials

Supplementary materials can be accessed at: http://www.mdpi.com/1420-3049/21/11/1505/s1.

## Abbreviations

BMGY	buffered minimal glycerol-complex medium
GalA	d-galacturonic acid
GAPDH	Glyceraldehyde-3-phosphate dehydrogenase
HPLC	High performance liquid chromatography
MD	Minimal dextrose
ORF	open reading frame
PBS	Phosphate buffered saline
RT-qPCR	real-time quantitative PCR
SDR	short-chain dehydrogenase/reductase
trun-OcUGlcAEs	Truncated OcUGlcAE proteins
UDP-Ara	UDP-l-arabinose
UDP-Gal	UDP-d-galactose
UDP-GalA	UDP-d-galacturonic acid
UDP-Glc	UDP-d-glucose
UDP-GlcA	UDP-d-glucuronic acid
UDP-Xyl	UDP-d-xylose
UGlcAE	UDP-d-glucuronic acid 4-epimerase
UGE	UDP-d-glucose 4-epimerase
UTR	untranslated region
UXE	UDP-d-xylose 4-epimerase
YPD	yeast extract–peptone–dextrose medium
