# Supplementary material for: cDNA Isolation and Functional Characterization of UDP-d-glucuronic Acid 4-Epimerase Family from Ornithogalum caudatum
Source: Molecules. 2016 Nov 9;21(11):1505. doi: 10.3390/molecules21111505 (PMC6273887; doi:10.3390/molecules21111505)
Supplement: Supplementary file 1 [file molecules-21-01505-s001.pdf]

Supplementary Materials: cDNA Isolation and Functional Characterization of UDP-D-glucuronic Acid 4-Epimerase Family from *Ornithogalum caudatum*

Sen Yin, Yu-Jia Sun, Ming Liu, Li-Na Li and Jian-Qiang Kong

Table S1. Strains and plasmids used in this study.

Strain/Plasmid	Description	Source
**Strain**		
*Trans*1-T1	*Escherichia coli*, F-ϕ80(*lac*Z)ΔM15Δ*lac*X74*hsd*R(r_K^-,m_K+)Δ*rec*A1398*end*A1*ton*A	TransGen, Beijing, China
*Trans*etta (DE3)	*E. coli*, F^-ompT*hsd*S_B($r_B^-m_B^-$)*gal dcm lac*Y1(DE3)pRARE(argU, argW,ilex,glyT,leuW,proL))Cam^r)	TransGen, Beijing, China
GS115	*Pichia pastoris*, His^-, Mut^+	Invitrogen, Carlsbad, CA, USA
**Plasmid**		
pEASY™-Blunt	General cloning vector, T7 promoter, f1 ori, Amp^r and Kan^r	TransGen, Beijing, China
pET-28a(+)	General expression vector, T7 promoter, f1 ori, Kan^r	Novagen, Madison, USA
pPIC3.5K	*Pichia pastoris* expression vector, AOX1 promoter	Invitrogen, Carlsbad, CA, USA
pGro7	Chaperone plasmid, araB promoter, Cm^r	
pEASY-OcUGlcAE1	*pEASY*™-Blunt derived plasmid containing *OcUGlcAE1* gene	This study
pEASY-OcUGlcAE2	*pEASY*™-Blunt derived plasmid containing *OcUGlcAE2* gene	This study
pEASY-OcUGlcAE3	*pEASY*™-Blunt derived plasmid containing *OcUGlcAE3*gene	This study
pET28aOcUGlcAE1(Δ1-116)	pET-28a(+) derived plasmid containing trun-*OcUGlcAE1* gene	This study
pET28aOcUGlcAE2(Δ1-136)	pET-28a(+) derived plasmid containing trun-*OcUGlcAE2* gene	This study
pET28aOcUGlcAE3(Δ1-128)	pET-28a(+) derived plasmid containing trun-*OcUGlcAE3* gene	This study
pPIC3.5KOcUGlcAE1	pPIC3.5K derived plasmid containing *OcUGlcAE1* gene	This study
pPIC3.5KOcUGlcAE2	pPIC3.5K derived plasmid containing *OcUGlcAE2* gene	This study
pPIC3.5KOcUGlcAE3	pPIC3.5K derived plasmid containing *OcUGlcAE3* gene	This study

Table S2. The putative TM helix of OcUGlcAE3 and other UGlcAEs from varied organisms.

UGlcAE	Possible TM Helix (aa~aa)
AtUGlcAE1	31~50
AtUGlcAE2	37~59
AtUGlcAE3	34~56, 85~107
ZmUGlcAE3	31~53
OsUGlcAE1	49~66,116~138
OsUGlcAE2	NO
OsUGlcAE3	31~53
OcUGlcAE3	36~58, 110~132

Table S3. Primers used in this research.

Primers	Sequences(5'-3')	Description
FGlcAE1-1	AGAGGGAAAAGAAAGATGAAG	Forward primer used for *OcUGlcAE1* amplification in the first round
RGlcAE1-1	CTCCTACCAATAACAAAAATCG	Reverse primer used for *OcUGlcAE1* amplification in the first round
FGlcAE1-2	ATGAGGATACTGGAGGAGGAGC	Forward primer used for *OcUGlcAE1* amplification in the second round
RGlcAE1-2	TTACAAATTCAAGCCCCCTCTC	Reverse primer used for *OcUGlcAE1* amplification in the second round
FGlcAE2-1	CTTGCAATCAATCATCAAAGAT	Forward primer used for *OcUGlcAE2* amplification in the first round
RGlcAE2-1	TTCGGGCGGCAGCAGAGC	Reverse primer used for *OcUGlcAE2* amplification in the first round
FGlcAE2-2	ATGCCGGCTCCATCGTCGTC	Forward primer used for *OcUGlcAE2* amplification in the second round
RGlcAE2-2	CTACTCTTTGTGCCCTCCTC	Reverse primer used for *OcUGlcAE2* amplification in the second round
FGlcAE3-1	CTCTATCTTTCTCTCTCTCTCT	Forward primer used for *OcUGlcAE3* amplification in the first round
RGlcAE3-1	GCAGCTCCCCCGTTCTTCC	Reverse primer used for *OcUGlcAE3* amplification in the first round
FGlcAE3-2	ATGGACGCGATGATCTCGCC	Forward primer used for *OcUGlcAE3* amplification in the second round
RGlcAE3-2	TCAGCTCGAGTTACTCCTCG	Reverse primer used for *OcUGlcAE3* amplification in the second round
F28aGlcAE1	GGTCGCGGATCCGAATTCATGGCCCTC AAGAAGCGCGGCGA	Forward primer used for pET28aOcUGlcAE1 (△1–116) construction
R28aGlcAE1	GAGTGCGGCCGCAAGCTTCAAATTCTT GCCCCCTCTCG	Reverse primer used for pET28aOcUGlcAE1 (△1–116) construction
F28aGlcAE2	GGTCGCGGATCCGAATTCATGAAGAAG CGAGGGGACGGTGT	Forward primer used for pET28aOcUGlcAE2 (△1–136) construction
R28aGlcAE2	GAGTGCGGCCGCAAGCTTCTCTTTGTGC CCTCCTCCTC	Reverse primer used for pET28aOcUGlcAE2 (△1–136) construction
F28aGlcAE3	GGTCGCGGATCCGAATTCATGTCGGCC GCCCTCAAGCGACG	Forward primer used for pET28aOcUGlcAE3 (△1–128) construction
R28aGlcAE3	GAGTGCGGCCGCAAGCTTGCTCGAGTT ACTCCTCGCAC	Reverse primer used for pET28aOcUGlcAE3 (△1–128) construction
F3.5kGlcAE1	ACTAATTATTCGAAGGATCCGCCACCA TGAGGATACTGGAGGAGGA	Forward primer used for pPIC3.5KOcUGlcAE1 construction
R3.5kGlcAE1	GCGCGGCCGCCCTAGGGAATTCTTACA AATTCTTGCCCCCTC	Reverse primer used for pPIC3.5KOcUGlcAE1construction
F3.5kGlcAE2	ACTAATTATTCGAAGGATCCGCCACCA TGCCGGCTCCATCGTCGTC	Forward primer used for pPIC3.5KOcUGlcAE2 construction
R3.5kGlcAE2	GCGCGGCCGCCCTAGGGAATTCCTACT CTTTGTGCCCTCCTC	Reverse primer used for pPIC3.5KOcUGlcAE2 construction

F3.5kGlcAE3	ACTAATTATTCGAAGGATCCGCCACCA TGGACGCGATGATCTCGCC	Forward primer used for pPIC3.5KOcUGlcAE3 construction
R3.5kGlcAE3	GCGCGGCCGCCCTAGGGAATTCTCAGC TCGAGTTACTCCTCG	Reverse primer used for pPIC3.5KOcUGlcAE3 construction
5′AOX1	GACTGGTTCCAATTGACAAGC	Forward primer used for PCR identification of pPIC3.5k derived plasmids
3′AOX1	GCAAATGGCATTCTGACATCC	Reverse primer used for PCR identification of pPIC3.5k derived plasmids
FOcUGLcAE1	CTTCAACCTTGGCAACACCT	Forward primer used for RT-qPCR analysis of *OcUGlcAE1*
ROcUGLcAE1	GGTCCTGGCGTAACTGATGT	Reverse primer used for RT-qPCR analysis of *OcUGlcAE1*
FOcUGLcAE2	TCCTCTCCTCCTCCTTCCTC	Forward primer used for RT-qPCR analysis of *OcUGlcAE2*
ROcUGLcAE2	GCCTGTCCCTCTTCAGTGAG	Reverse primer used for RT-qPCR analysis of *OcUGlcAE2*
FOcUGLcAE3	ACATTGCTGGCCTTGTTACC	Forward primer used for RT-qPCR analysis of *OcUGlcAE3*
ROcUGLcAE3	CCGTACACCGTGAAGAACCT	Reverse primer used for RT-qPCR analysis of *OcUGlcAE3*
FGAPDH2	ACTTGGTGTCCACCGACTTC	Forward primer used for RT-qPCR analysis of *GAPDH1*
RGAPDH2	ATTCGTTGTCGTACCAAGCC	Reverse primer used for RT-qPCR analysis of *GAPDH1*

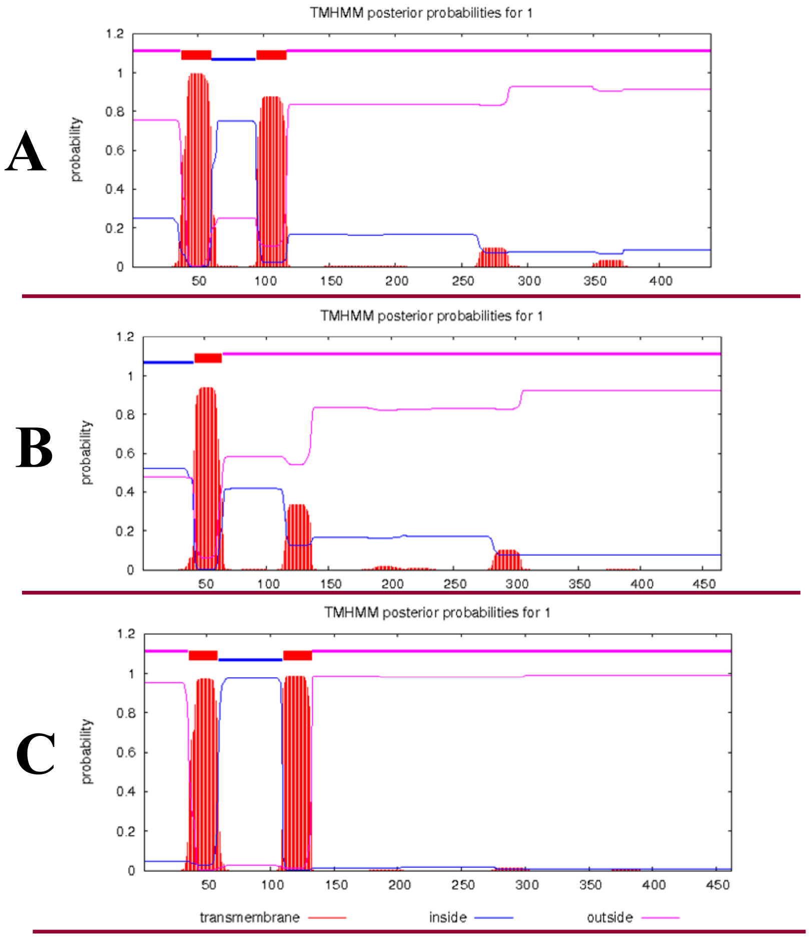

Figure S1. The predicted transmembrane helices of OcUGlcAE1 (**A**); OcUGlcAE2 (**B**) and OcUGlcAE3 (**C**).

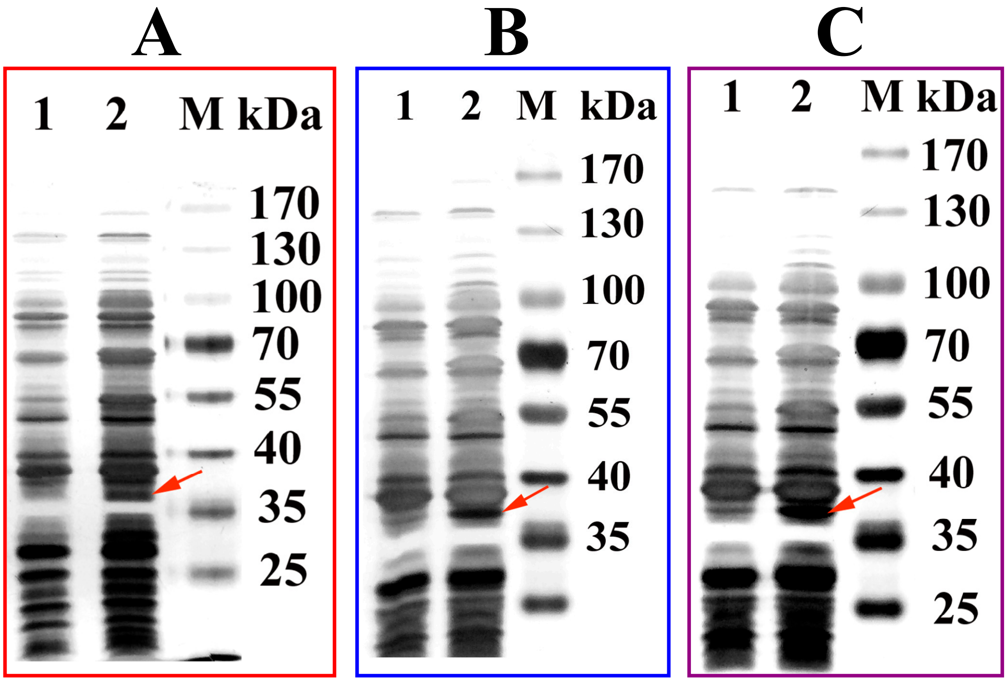

Figure S2. SDS-PAGE analyses of total soluble proteins isolated from *E. coli* cells expressing OcUGlcAE1 (Δ1–116) (**A**); OcUGlcAE2 (Δ1–136) (**B**) or OcUGlcAE3 (Δ1–128) (**C**). Lanes 1 and 2 referred to the total extract of cells harboring the empty vector and the recombinant vector, respectively. The arrows indicated the overproduced proteins

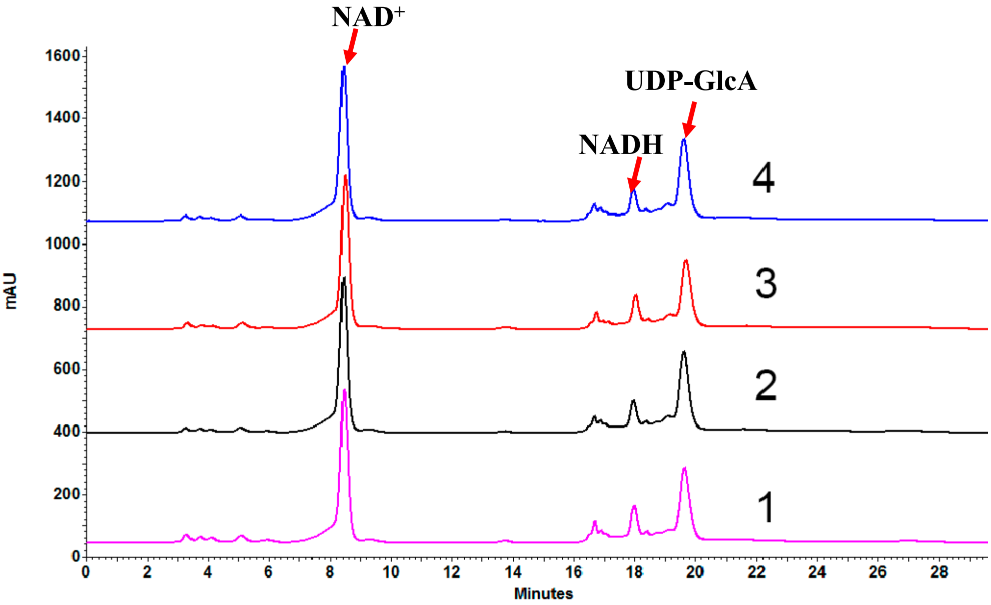

Figure S3. HPLC profiles of reaction mixtures generated by *E. coli* expressing the empty vector alone (**1**), pET28aOcGlcAE1 (Δ1–116) (**2**), pET28aOcGlcAE2 (Δ1–136) (**3**) or pET28aOcGlcAE3 (Δ1–128) (**4**) in the presence of UDP-GlcA and NAD$^+$.

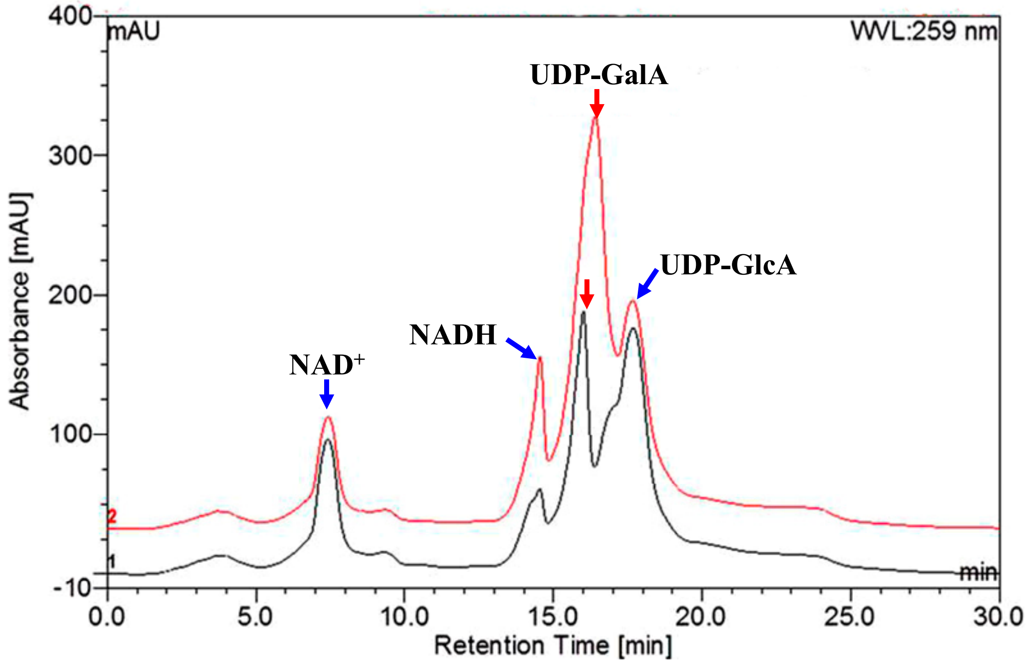

Figure S4. HPLC profiles of OcUGlcAE3-catalyzed reaction mixtures co-injected with (**2**) or without UDP-GalA (**1**). The red arrows stand for UDP-GalA.

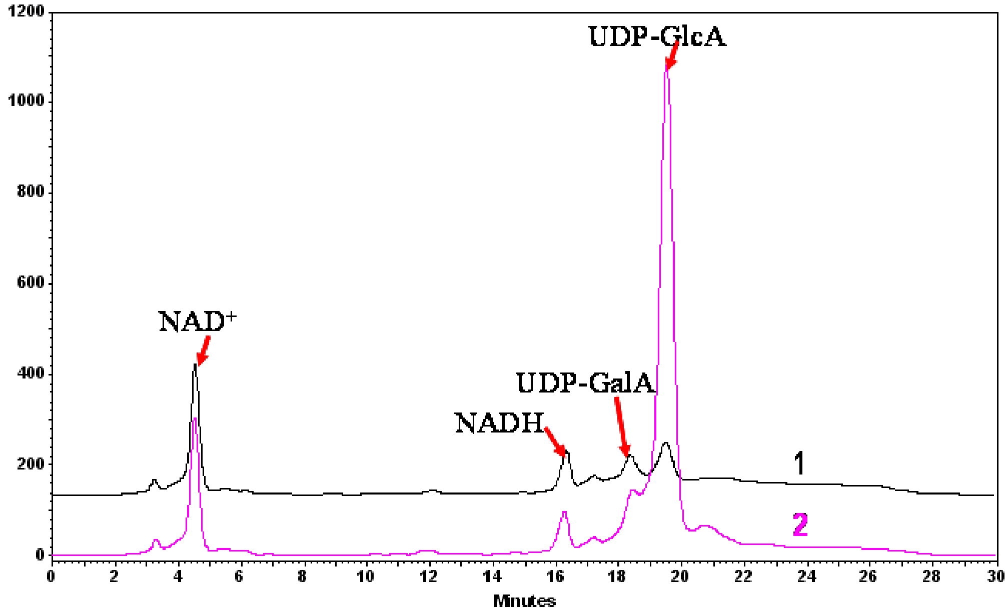

Figure S5. HPLC profiles of OcUGlcAE3-catalyzed reaction mixtures co-injected with (**1**) or without UDP-GlcA (**2**).

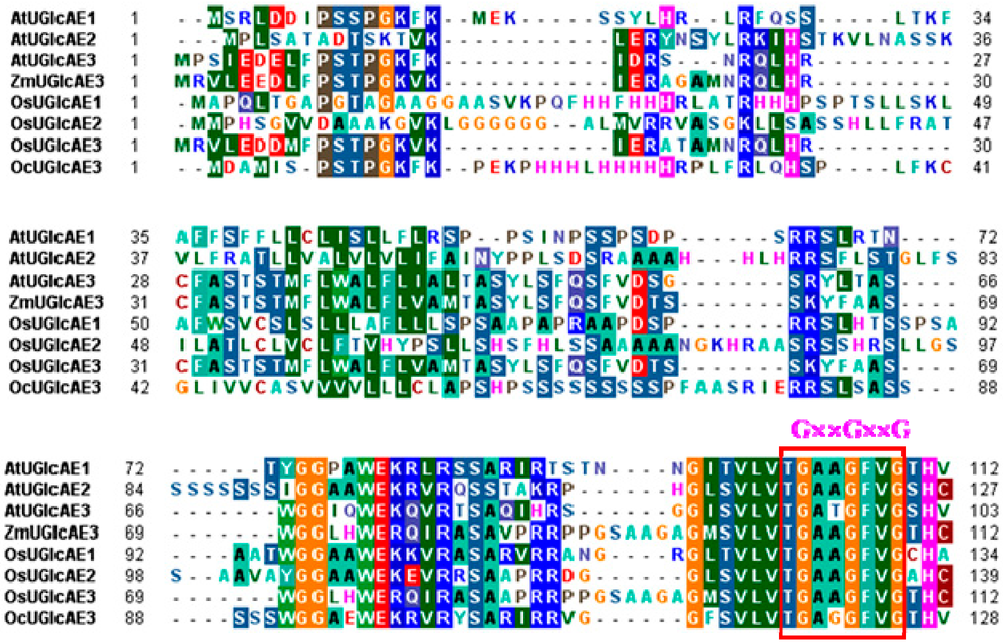

Figure S6. N-terminal alignment of OcUGlcAE3 and other UGlcAEs from varied organisms. The red box represents the G××G××G motif.
